# Analysis of the effects of related fingerprints on molecular similarity using an eigenvalue entropy approach

**DOI:** 10.1186/s13321-021-00506-2

**Published:** 2021-03-23

**Authors:** Hiroyuki Kuwahara, Xin Gao

**Affiliations:** Computational Bioscience Research Center (CBRC), King Abdullah University of Science and Technology (KAUST), Thuwal, 23955 Saudi Arabia

**Keywords:** Structure-activity relationship, Similarity-based virtual screening, 2D fingerprint, Unsupervised feature selection, Chemoinformatics

## Abstract

Two-dimensional (2D) chemical fingerprints are widely used as binary features for the quantification of structural similarity of chemical compounds, which is an important step in similarity-based virtual screening (VS). Here, using an eigenvalue-based entropy approach, we identified 2D fingerprints with little to no contribution to shaping the eigenvalue distribution of the feature matrix as related ones and examined the degree to which these related 2D fingerprints influenced molecular similarity scores calculated with the Tanimoto coefficient. Our analysis identified many related fingerprints in publicly available fingerprint schemes and showed that their presence in the feature set could have substantial effects on the similarity scores and bias the outcome of molecular similarity analysis. Our results have implication in the optimal selection of 2D fingerprints for compound similarity analysis and the identification of potential hits for compounds with target biological activity in VS.

## Introduction

Virtual screening (VS) is a computational approach that is widely used as a cost-effective alternative to the traditional high-throughput screening for the selection of initial hits in a search for drugs with a given biological activity [1, 2]. The foundation of similarity-based VS is structure-activity relationship (SAR), a concept in which molecules with similar structures are destined to have similar biological activities. In such VS applications, thus, the quantification of structural similarity of molecules is a crucial step. To quantify the structural similarity of a pair of molecules, the Tanimoto similarity measure is commonly applied to fingerprint features based on their two-dimensional (2D) structures. These 2D fingerprints represent each molecule as a binary (0 or 1) vector characterizing the absence or the presence of specific properties of its 2D structure. Although this feature representation is simple, it has been reported to be more effective than those using more complex features such as 3D structural patterns [3, 4].

There are libraries of predefined 2D chemical fingerprint dictionaries available to represent molecules as binary vectors [5]. Among the most commonly used fingerprint schemes for similarity quantification is molecular access system (MACCS) [6], which was reported to cover many useful 2D features for virtual screening [7]. While these predefined fingerprint dictionaries are easy to use, previous studies demonstrated that the selection of relevant 2D fingerprints from the original set resulted in better performance [8–10]. These feature selection methods typically focus on supervised machine learning settings in which to select a subset of relevant 2D fingerprints that intend to enhance the generality to discriminate chemical compounds with a given biological activity against those without. For example, Nisius, et al. ranked 2D fingerprints by applying the Kullback-Leibler divergence to each fingerprint to quantify its asymmetric usage between the active compound class and the inactive one [11]. Given the nature of drug discovery, however, these supervised feature selection approaches inevitably face a challenging class imbalance problem in practice as available compounds with the target biological activity is most likely very scarce. That is, had the number of target bioactive compounds been large enough to begin with, a pipeline to discover more of the same would not have probably warranted a large cost of investment.

**Fig. 1:**
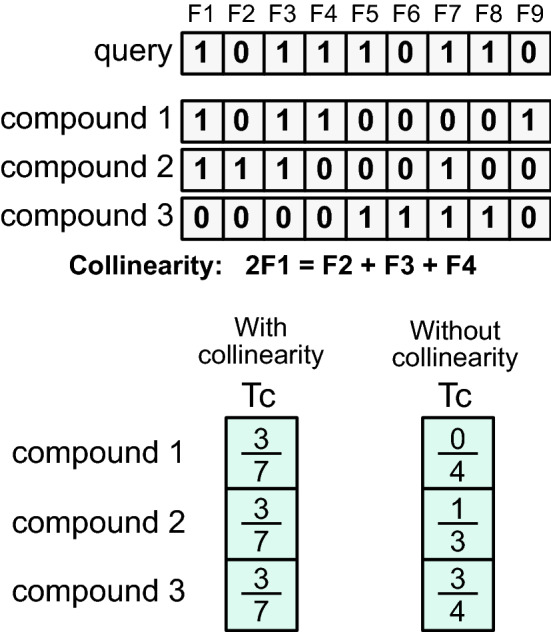
An illustrative example for the effects of related fingerprints on similarity measures. A hypothetical fingerprint scheme with nine bit keys ($F_1$ to $F_9$) is used to represent small molecules in a hypothetical compound dataset. The fingerprint matrix of this dataset is found to have a perfect multicollinearity in the first four features with $2\,F_1 = F_2 + F_3 + F_4$. The similarity of a query compound against three compounds is computed using Tanomoto coefficient (Tc) with and without this collinearity. For the results without the collinearity, the Tanimoto coefficient without the first four features ($F_1$ to $F_4$) is shown

Here, we focus on a different issue in the combination of 2D fingerprints and analyze the effects of related fingerprints on the quantification of molecular similarity using eigenvalue-based entropy. The eigenvalue-based entropy was introduced by Alter et al. [12] to indicate the weight distribution of gene expression eigenvectors for analysis of temporal gene expression patterns. Varshavsky et al. [13] developed an unsupervised feature selection method that ranks each feature by measuring its contribution to the eigenvalue-based entropy. We defined the relatedness of each 2D fingerprint based on the degree to which the shape of the eigenvalue distribution of the feature matrix is changed. And, by using the eigenvalue-based entropy as the scaler value to indicate the distribution of eigenvalues, we determined related 2D fingerprints. Thus, we defined a related 2D fingerprint as a feature that has a (quasi) linear relationship with some other fingerprints in the feature set regardless of its relevance and importance for the discriminability. As illustrated in Fig. 1, the presence of such related fingerprints can inflate or deflate similarity scores, potentially changing the outcome of molecular similarity analysis and VS.

In this paper, we applied MACCS and Pubchem fingerprint schemes to human metabolite and drug compound datasets and identified many fingerprints as related ones. While the effects of related fingerprints depended on various factors such as query compounds, the general trend of the effects of the presence of related 2D fingerprints was found to mildly lower overall similarity scores and some of these negative effects were found to be substantial. Our analysis demonstrated that these effects can pose challenges in ranking similar compounds and qualitatively change the outcome of VS.

## Methods

### Datasets

From Human Metabolome Database (HMDB) [14], we retrieved 2D structure data for 25,376 metabolites on September 5, 2019. With a filtering for the metabolites found in blood with the metabolite status being “Detected and Quantified,” we further obtained the information about 3,202 metabolites for the blood specimen.

From DrugBank (version 5.1.7) [15], we downloaded the dataset for aproved drugs with 2,636 entries. After preprocessing for unique and valid SMILES data, we obtained 2,466 drug compounds for the DrugBank dataset.

### Molecular similarity measure

We used the implementation of CDK (version 2.3) [16] to compute 166-bit MACCS and 881-bit Pubchem fingerprint vectors. To measure the similarity of a pair of compounds, *a* and *b*, we computed the Tanimoto coefficient of their *l*-bit fingerprint vectors, $v_a$ and $v_b$ as follows:

1 $$\begin{aligned} sim(a,b) = \frac{\sum _{i=1}^{l}{v_a(i) v_b(i)}}{\sum _{i=1}^{l}{v_a(i) + v_b(i) - v_a(i) v_b(i)}}, \end{aligned}$$

where *v*(*i*) represents the *i*-th element of vector *v*.

### Eigenvalue-based entropy

Let *A* be an *m* by *n* matrix. Then, an *n* by *n* symmetric matrix $A^T A$ is positive semidefinite and has real eigenvalues $\lambda _1 \ge \lambda _2 \ge \cdots \ge \lambda _n \ge 0$. By defining $q_j$ to be the *j*-th normalized eigenvalue $q_j = \lambda _j / \sum _{k=1}^{n}{\lambda _k}$, we computed a single value that indicates the complexity of the distribution of eigenvalues with the normalized entropy of eigenvalues [12] as follows:

2 $$\begin{aligned} H = -\frac{1}{\log {(n)}}\sum _{j=1}^{n}{q_j \log {(q_j)}}. \end{aligned}$$

This entropy ranges from 0 to 1, with 0 indicating that the dataset can be constructed based on a single eigenvector and 1 indicating that each eigenvector has an equal contribution to the dataset. Eigenvalues were computed using the *svd* function in R.

### Eigenvalue-based fingerprint contribution measure

Suppose we have *m* compounds, each of which is expressed with *n*-bit 2D fingerprints. That is, we have an *m* by *n* matrix *A* whose element $a_{i,j}$ represents the value of the *j*-th fingerprint for the *i*-th compound. Let $A[-i]$ be an *m* by *n* matrix that has all but the *i*-th column of *A*, with the *i*-th column replaced by a zero column. We computed the contribution of the *i*-th ($1 \le i \le n$) fingerprint, $h_i$ as $h_i = H(A[-i])$ where *H*(*M*) is the eigenvalue-based entropy of matrix *M* given by Equ. 2. Note that, because we can first compute *n*-by-*n* matrix from $A^T A$, the computation of each fingerprint entropy depends on the number of the fingerprints and not on the number of compounds, which is presumed to be very large.

### Contribution of related fingerprints to the similarity score

Suppose we have two *l*-bit fingerprint vectors $v_a$ and $v_b$. Further suppose that two *k*-bit fingerprint vectors $u_a$ and $u_b$ are subvectors of $v_a$ and $v_b$, respectively, that represent their related fingerprints. To measure the contribution of the related fingerprints to the Tanimoto coefficient, we compute two scores: the contribution to the union set and the contribution to the intersecting set. The contribution to the union set of $v_a$ and $v_b$ is defined to be the ratio of the union set of the related fingerprint vectors to the union set of the original fingerprint vectors as follows:

3 $$\begin{aligned} \frac{\sum _{i=1}^{k}{u_a(i) + u_b(i) - u_a(i) u_b(i)}}{\epsilon + \sum _{i=1}^{l}{v_a(i) + v_b(i) - v_a(i) v_b(i)}}, \end{aligned}$$

while the contribution to the intersecting set of $v_a$ and $v_b$ is defined to be the ratio of the intersecting set of the related fingerprint vectors to the intersecting set of the original fingerprint vectors as follows:

4 $$\begin{aligned} \frac{\sum _{i=1}^{k}{u_a(i) u_b(i)}}{\epsilon + \sum _{i=1}^{l}{v_a(i) v_b(i)}}, \end{aligned}$$

where $\epsilon$ is a small constant (e.g., $10^{-10}$) to avoid the division by zero.

## Results

### Presence of highly correlated fingerprints

MACCS keys are 166-bit 2D structure fingerprints that are commonly used for the measure of molecular similarity. Because each bit is either on (i.e., 1) or off (i.e., 0), MACCS 166 keys can represent more than $9.3 \times 10^{49}$ distinct fingerprint vectors. After removing 454 entries with duplicate canonical SMILES strings, we generated 24,922 MACCS fingerprint vectors using the metabolite data we obtained from HMDB [14] (see "Methods"). After filtering out duplicates, we ended up with 3,125 unique fingerprint vectors. On average, thus, 8 metabolites were represented by the same MACCS fingerprint vector, indicating a high degree of collisions. The high level of collided metabolites suggests the possibility that many MACCS keys describe related 2D substructure characteristics.

**Fig. 2:**
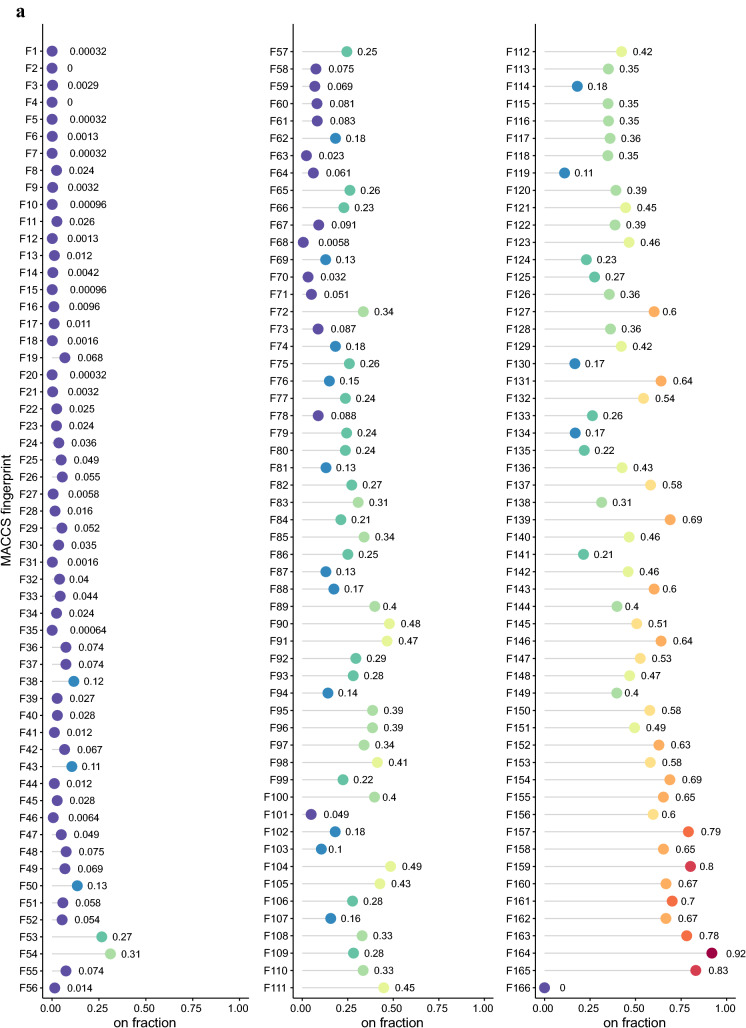

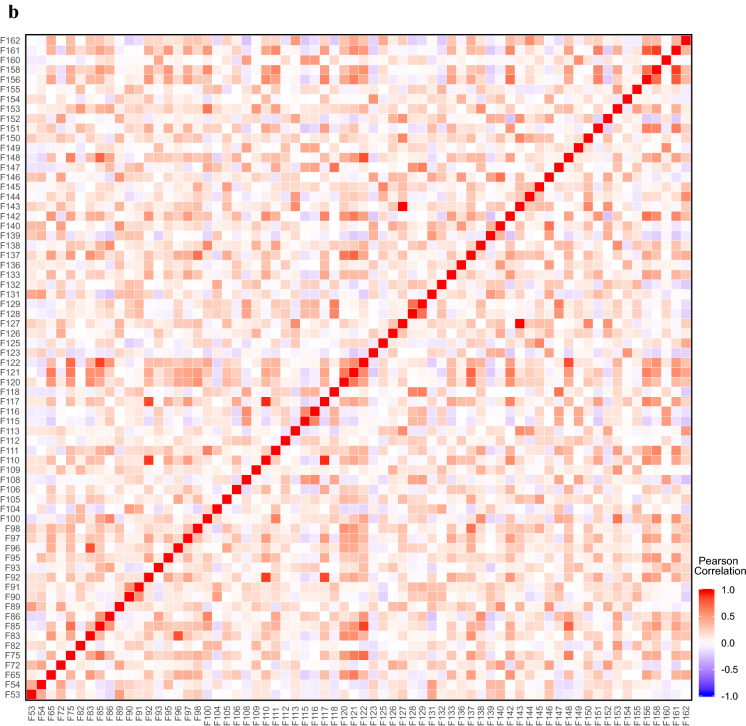
Fingerprint usage patterns of MACCS 166 keys on HMDB metabolite dataset. **a** The on-bit count of each key. **b** The pairwise Pearson’s correlation coefficient value for each pair of 68 MACCS keys with moderate on-bit counts

To analyze the use of each MACCS key, we first counted the occurrence of on bit for each key in the 3,125 unique fingerprint vectors (Fig. 2a). We found that 39% of the 166 MACCS keys are on (i.e., 1) for fewer than 10% of the fingerprint vectors, while only 1 key is on for more than 90% of the vectors. This skewed use of molecular fingerprints ($\gamma _1 = 0.777$) indicates that many fingerprint bits are set to be off (i.e., 0) in most of the vectors, resulting in highly similar usage patterns. However, since Tanimoto coefficient, the most commonly used 2D fingerprint-based similarity measure, does not consider the off bits (see "Methods"), its similarity analysis of these HMDB metabolites may not be influenced by the fingerprints with many off bits.

We next analyzed the association of MACCS keys whose on-bit counts are more moderate and whose effects on similarity measure are assumed to be more profound. To this end, we focused on a subset of the MACCS keys whose on-bit counts are in a range between 25% and 75% of the total number of the unique vectors. We obtained 68 MACCS keys that satisfied this constraint and computed their pairwise correlation coefficient values (Fig. 2b). We found that a large fraction of the pairs (73%) had positive correlation ($r \ge 0$). Out of 2,278 fingerprint pairs, while none had strong negative correlation ($r \le -0.5$), 105 had strong positive correlation ($r \ge 0.5$). Among these positively correlated pairs, the 127th and the 143rd fingerprints, both of which had 1,875 on-bit counts, had the perfect positive correlation, suggesting that 2D structures characterized by these two fingerprints are highly related. Although the correlation coefficient can capture only a limited type of related fingerprints, these results suggest the prevalence of related fingerprints in the predefined 2D fingerprint dictionaries.

### Characterization of related fingerprints

We next sought to analyze the extent to which more general types of related fingerprints were present in the MACCS and Pubchem fingerprint dictionaries. To this end, we gathered 3202 metabolites found in the blood specimen from the HMDB metabolite dataset and filtered out compounds with duplicate 2D structures, duplicate fingerprint vectors, and all-zero MACCS fingerprint vectors. In addition, we removed each compound whose MACCS fingerprint vector has off bits for more than 90% of the fingerprints.

With this data preprocessing, we selected 1023 metabolites that have unique fingerprint vectors. The 1023 by 166 matrix formed with the MACCS fingerprints had the rank of 144, where the column represents the fingerprints and the row represents the metabolite (see "Methods"), indicating that the pattern of $\sim 15\%$ of MACCS fingerprints can be completely captured by the rest. The Pubchem fingerprints resulted in a 1023 by 881 fingerprint matrix that had the rank of 377, indicating even more pronounced effects of rank deficiency with more than half of the fingerprints completely characterized by linear combinations of 377 fingerprints.

**Fig. 3:**
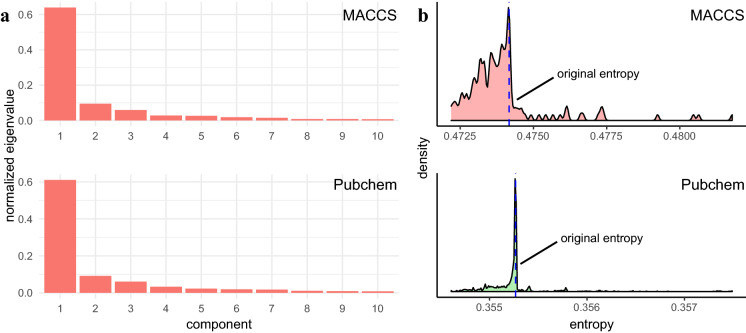
Eigenvalue-based analysis of MACCS and Pubchem fingerprint matrices.** a** Normalized eigenvalues of the first 10 components for MACCS and Pubchem fingerprint matrices. **b** The distribution of the eigenvalue-based entropy for MACCS and Pubchem fingerprints

To assess the degree of related fingerprints, we defined the relatedness using the eigenvalue-based entropy (see "Methods"). This eigenvalue-based entropy measure indicates the shape of the eigenvalue distribution [12], with its value ranging from 0 to 1 where a lower value indicates that the matrix can be reconstructed with a linear combination of a smaller number of eigenvectors. The distribution of the normalized eigenvalues for the MACCS and Pubchem fingerprint matrices shows that the first component has $> 6$ times higher weight than the second one in both (Fig. 3a), indicating that their entropy values must be lower. Indeed, the entropy values of the original MACCS and Pubchem matrices were 0.474 and 0.355, respectively. To measure the relatedness of the *i*-th fingerprint with the other fingerprints, we computed the change in the entropy between the original fingerprint-feature matrix and the feature matrix without the *i*-th fingerprint (see "Methods"). This can indicate the contribution of the *i*-th fingerprint to shaping the eigenvalue distribution, which, in turn, allows us to evaluate the degree to which the *i*-th fingerprint is linearly related to some other fingerprints. Figure 3b shows the distribution of the fingerprint entropy values for both the MACCS and Pubchem schemes. We found a high peak at the entropy value of the original fingerprint-feature matrix, with many fingerprints having their entropies in near the original one, indicating that these fingerprints do not contribute much to the eigenvalue distribution and are highly related to some other fingerprints.

**Fig. 4:**
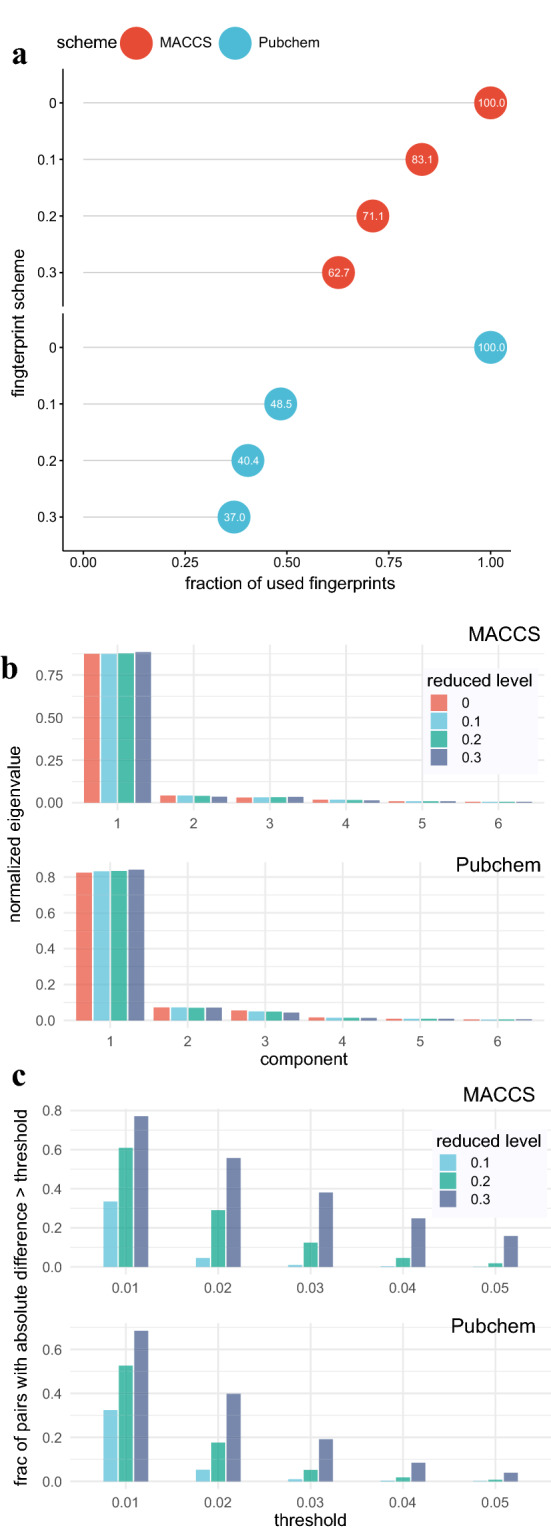
Comparison of the Tanimoto similarity score based on different reduction levels. **a** The fraction of used fingerprints with respect to given reduced levels for MACCS and Pubchem fingerprints. The reduced level 0 indicates the fraction for the original fingerprints. **b** The comparison of the first 6 principal components with respect to different reduction levels. **c** The comparison of the fraction of metabolite pairs with the absolute similarity score difference between the original set of fingerprints and a given reduced set of fingerprints exceeding specified threshold values

By measuring the relatedness based on the distance between the original entropy and the entropy for each fingerprint, we selected related fingerprints from the original MACCS and Pubchem fingerprint dictionaries. Let $h_0$ and $h_i~(1 \le i \le n)$ be the eigenvalue-based entropy of the original feature matrix and for the *i*-th fingerprint, respectively. Then, we selected the *i*-th fingerprint as a related feature if $h_i$ satisfies the following condition:

5 $$\begin{aligned} |h_i - h_0 |< z \sqrt{\frac{\sum _{j=1}^n{\left( h_j - h_0 \right) }^2}{n}} , \end{aligned}$$

where *z* is a reduced-level threshold parameter, which was set to 0.1, 0.2, and 0.3 in this study. Based on this approach, we found that many fingerprints in the MACCS and Pubchem dictionaries are related (Fig. 4a). With the reduced-level threshold being 0.1, 0.2, and 0.3, we identified 28, 48, 62 related fingerprints in MACCS and 454, 525, and 555 in Pubchem, respectively. A larger fraction of the related fingerprints identified in the Pubchem scheme with the low threshold value was expected given that the distribution of its fingerprint entropies had a higher density of the fingerprints near the original entropy.

### Effects of related fingerprints on molecular similarity scores

Using the related fingerprints identified with the eigenvalue-based entropy approach, we set out to examine their effects on the similarity score. To this end, we first constructed 1023 by 1023 similarity matrix by computing the Tanimoto coefficient for each metabolite pair and generated the normalized eigenvalues of similarity matrices (Fig. 4b). The comparison of the first six components suggests that the similarity matrices computed from fingerprint sets with various reduction levels in both MACCS and Pubchem schemes are similar. Next, we measured the absolute difference of 522,753 distinct metabolite pairs between the original fingerprint set and reduced fingerprint sets. We found that the difference increased as the reduced level threshold increased in both MACCS and Pubchem fingerprint dictionaries (Fig. 4c). While both fingerprint schemes had quantitatively similar levels of absolute differences with the reduced level at 0.01, the difference became wider as the threshold increases particularly in MACCS, suggesting the effects of removing related fingerprints were greater in the MACCS scheme even though a higher fraction of fingerprints were removed in the Pubchem scheme.

To further analyze the effects of related fingerprints on the Tanimoto similarity scores, we grouped the metabolites in the blood specimen into four classes: drug, microbial, plant, and endogenous using the metabolite annotation retrieved from HMDB. In each of these four categories, we computed the average of the pairwise Tanimoto similarity scores. The results show that the average similarity scores from the reduced fingerprint sets are quantitatively close to those from the original fingerprint sets (Table 1). Similarity scores from the reduced fingerprint sets were found to be marginally higher than those from the original fingerprint sets. In other words, the inclusion of related fingerprints had negative effects and slightly decreased the Tanimoto similarity score.

**Table 1:** The average Tanimoto similarity score for five classes of metabolites in the blood specimen for the MACCS and Pubchem fingerprint schemes with different reduction levels

Scheme	Level	Drug	Microbial	Plant	Endogenous	All
MACCS	0	0.3008	0.3531	0.3662	0.3211	0.3142
MACCS	0.1	0.2990	0.3489	0.3696	0.3190	0.3122
MACCS	0.2	0.2944	0.3536	0.3723	0.3188	0.3118
MACCS	0.3	0.3013	0.3578	0.3785	0.3211	0.3149
Pubchem	0	0.3048	0.3323	0.3873	0.2967	0.2968
Pubchem	0.1	0.3104	0.3390	0.3922	0.3012	0.3016
Pubchem	0.2	0.3167	0.3384	0.3974	0.3043	0.3053
Pubchem	0.3	0.3217	0.3395	0.4010	0.3071	0.3085

**Fig. 5:**
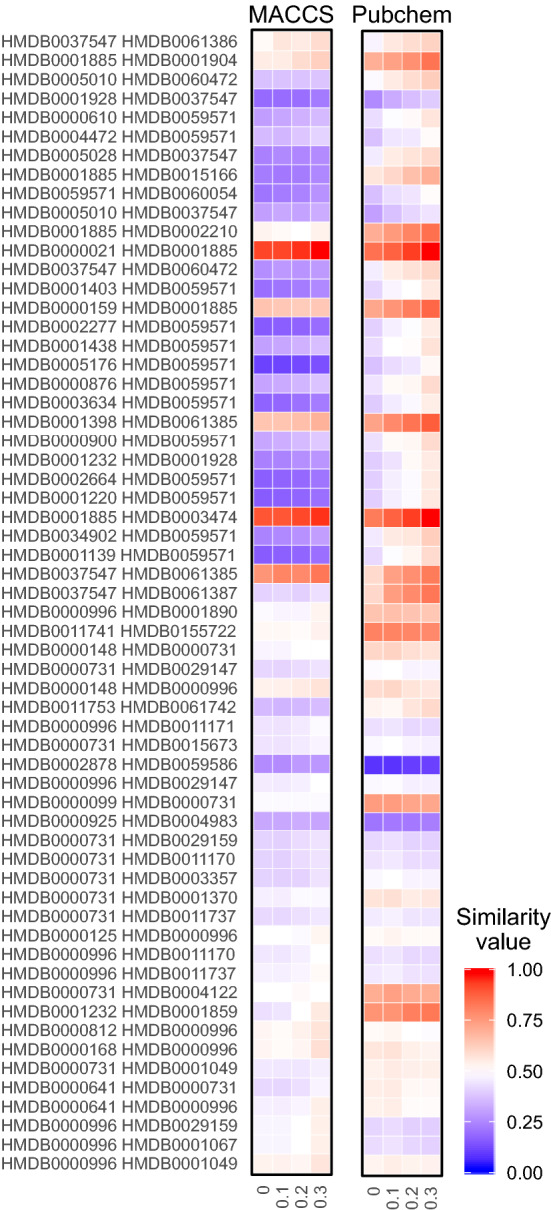
Illustration of 60 metabolite pairs with high levels of changes in Tanimoto similarity measures. Heatmap showing the similarity scores of 60 metabolite pairs based (y-axis) on given levels of reduced fingerprint sets (x-axis). From the MACCS and Pubchem fingerprint dictionaries, 30 pairs are selected from each based on the difference between the original set of fingerprints and a reduced set of fingerprints with reduced level 0.3

To characterize the significance of the negative effects of the related fingerprints on individual compound pairs, we identified the pairs with the 30 largest absolute differences in the similarity score computed with two reduced-level thresholds 0 and 0.3 for each of the MACCS and Pubchem fingerprint schemes. From the comparison of the similarity scores for different threshold levels in these 60 compound pairs, the negative effects of the related fingerprints on the similarity score were found to be prevalent (Fig. 5). Indeed, the similarity scores using the reduced fingerprints with the threshold 0.3, for example, demonstrated strong association between the related fingerprints and the negative effects on the similarity scores ($p < 10^{-22}$ with two-sided exact binomial test).

### Effects of related fingerprints for different query compounds

Next, we set out to examine the extent to which related fingerprints can impact the analysis of similar compounds for different query compounds. To this end, we used the drug compound data from DrugBank 5.1.7 (see "Methods") and generated the fingerprint vectors of the 2,466 drugs using MACCS and Pubchem fingerprint schemes. We performed the eigenvalue-based entropy approach on the dataset with 0.3 as the reduced-level threshold, identifying 34 and 470 fingerprint features as related features for the MACCS and Pubchem schemes, respectively. We then randomly selected 10 drugs as the query compounds and measured their structural similarity against the other 2,456 compounds using the Tanimoto coefficient. To analyze the contribution of the related fingerprint features for each query compound, we used the compounds with the 50 highest similarity scores. Because the Tanimoto coefficient of a pair of fingerprint vectors is the ratio of the size of their intersecting set to that of their union set, we measured the contribution to the intersecting set and the union set, separately (see "Methods"). When the contribution of the related fingerprints to the intersecting set is higher than the union set, they have positive effects on the Tanimoto coefficient. By contrast, when their contribution to the intersecting set is lower than the union set, they have negative effects on the Tanimoto coefficient.

**Fig. 6:**
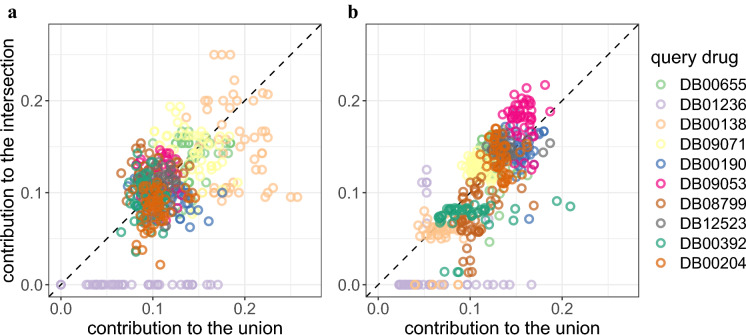
Contribution of related fingerprints to the similarity score for 10 randomly selected query compounds in DrugBank. The scatterplot shows two contribution measures from the Tanimoto coefficient (the ratio of the intersecting set to the union) of the drug compounds with the 50 highest similarity scores for each query compound. The x-axis shows the contribution of the related fingerprints to the union set, while the y-axis shows the contribution to the intersecting set. The related fingerprints are defined to be the removed ones based on the reduced level 0.3. **a** MACCS scheme. **b** Pubchem scheme

The contribution of related fingerprints to the Tanimoto coefficient was found to vary among different compound pairs as well as between MACCS and Pubchem (Fig. 6). Although, in both schemes, the related fingerprints had positive and negative effects on similarity scores of drug pairs, their effects to the union set were more significant and prevalent in the MACCS fingerprint scheme. In addition, consistent with the analysis on the HMDB dataset, stronger effects of the related fingerprints were found in those pairs with higher contribution to the union set for both MACCS and Pubchem schemes (i.e., those with negative effects).

Interestingly, the contribution of the related fingerprints displayed clustering patterns based on the query compounds. With Sevoflurane (DB01236) as the query drug, for example, the related fingerprints contributed minimal on the intersecting set compared with the union set for both MACCS and Pubchem, indicating that the related fingerprints had stronger negative effects on the similarity scores for this drug. For Ibrutinib (DB09053), while they contributed to both the intersecting and the union sets more than 10%, the related fingerprints influenced the intersecting set more, indicating that they had positive effects on the similarity score. These results indicates that the impact of related fingerprints can depend strongly on query compounds, suggesting that the presence of related fingerprints can give rise to bias in similarity scores, potentially making fair analysis of similar compounds for various query compounds challenging.

### Effects of related fingerprints on drug similarity accuracy

Although our results showed the effects of related fingerprints on molecular similarity analysis based on the Tanimoto coefficient, it is not clear if those effects can lead to qualitatively significant changes in structural similarity-based molecule screening. To analyze potential effects of related fingerprints in such SAR-based analysis, we used a dataset consisting of 100 drug compound pairs from DrugBank 3.0 [17] that 143 experts analyzed to provide their yes or no binary decisions about the structural similarity [18]. To use this dataset as the correct reference for similar compounds, we selected a subset of the 100 pairs whose similarity was supported by at least 80% of the experts, resulting in 33 pairs of similar compounds. The Tanimoto similarity scores of these compound pairs were computed using the original fingerprint set as well as the reduced one from the DrugBank 5.1.7 dataset, with the reduced-level threshold set to 0.3.

**Table 2:** Summary of the results from 33 pairs of similar compounds with high consensus from 143 experts using the original fingerprint set and the reduced fingerprint set

Scheme	Fingerprints	Mean	Std. dev.	$\ge 0.8^a$	$\ge 0.7^b$	min $\hbox {sim}^c$
MACCS	Original	0.8724	0.1308	24	31	0.4686
MACCS	$\hbox {reduced}^d$	0.8794	0.1229	28	31	0.5000
Pubchem	Original	0.9163	0.0809	29	32	0.6970
Pubchem	Reduced	0.9187	0.0750	29	33	0.7402

$^a$The number of instances in which the similarity score is greater than or equal to 0.8.

$^b$The number of instances in which the similarity score is greater than or equal to 0.7.

$^c$The minimum similarity score among the 33 pairs.

$^d$The reduced fingerprint set using 0.3 as the threshold

We first analyzed the performance of each fingerprint set by computing five measures: the mean of the similarity scores, the standard deviation of the scores, the number of pairs whose similarity scores are $\ge 0.8$, the number of pairs whose similarity scores are $\ge 0.7$, and the minimum similarity score (Table 2). We decided to focus on the number of positives because in virtual screening molecular similarity is used to filter out incompatible compounds and to generate initial hits with potentially similar bioactive properties in order to capture them in follow-up screenings [1]. That is, in the filtering for the initial hits, as long as true positives are included, the number of false positives is not as important.

The reduced fingerprint set resulted in an increase in the average similarity scores and a decrease in the variance of the similarity scores mildly yet consistently in both MACCS and Pubchem schemes. An increase in the overall similarity score is consistent with the results from the HMDB dataset, while a decrease in the variance can be explained by the enhanced generalization achieved with the removal of related fingerprints. The number of pairs with high similarity sores also increased by removing related fingerprints; in the MACCS scheme, the original fingerprint set and the reduced fingerprint set with the threshold 0.03 correctly predicted 73% and 85% of the reference pairs with the positive-calling threshold of 0.8, while in the Pubchem scheme, they correctly predicted 97% and 100% of the pairs with the positive-calling threshold of 0.7. Furthermore, in both fingerprint schemes, the reduced fingerprint set resulted in $>6\%$ increase in the minimum similarity score among the 33 reference pairs, indicating that the filtering of related fingerprints was able to improve the similar compound search more inclusively.

**Fig. 7:**
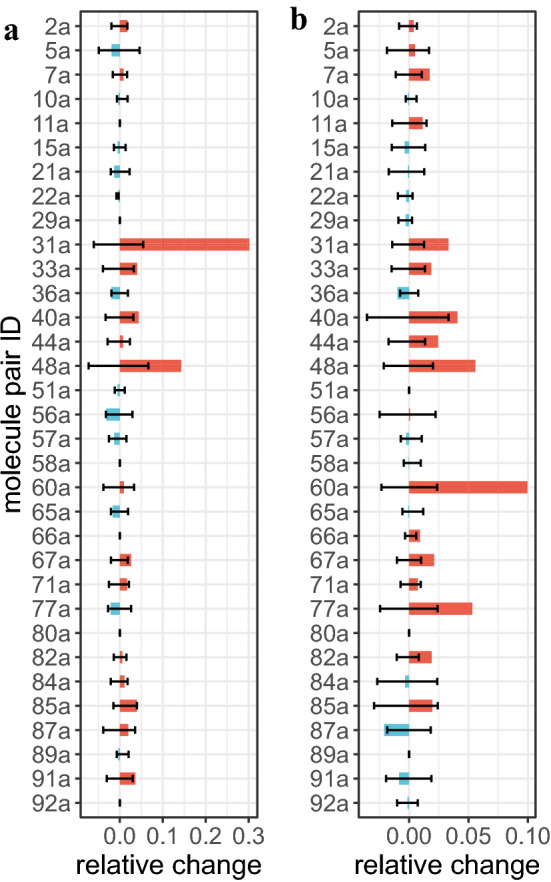
Relative changes of the Tanimoto similarity measure with the fingerprints in the reduced level 0.3 with respect to the one with the original fingerprints. The relative similarity changes are shown for the 33 similar-compound pairs with a high consensus by the 143 experts ($\ge 80\%$). The error bars indicate the 1st and the 3rd quartiles of 10,000 Tanimoto coefficients computed with random pruning of the original fingerprints. These randomly selected fingerprint vectors have the same length as the one for the the reduced level 0.3. **a** MACCS scheme. **b** Pubchem scheme

To better understand the significance of the results from the reduced fingerprint set, we generated 10,000 sets of reduced fingerprints by randomly pruning the original fingerprint set to have the same size as the one with the reduced-level threshold 0.03. The comparison revealed that the filtering of related fingerprints had a tendency to substantially increase the similarity scores of a number of pairs, while the random pruning did not show such directionality bias (Fig. 7). To further analyze significant changes from the filtering of related fingerprints, we computed the p-value with the null hypothesis that the filtering of related fingerprints is the same as the random pruning. We obtained two and four pairs with significant changes ($p < 0.05$) from the MACCS and Pubchem schemes, respectively, and all of these pairs resulted in an increase in their similarity scores with the removal of related fingerprints. These results indicate that related fingerprints are likely to contribute to the union set more significantly and that the filtering of such fingerprint features can increase the Tanimoto coefficient of structurally similar compound pairs.

## Conclusions

In this study, we defined related fingerprints to be those that do not contribute to the shape of the eigenvalue distribution of the original fingerprint feature matrix and thus are thought to possess a high degree of multicollinearity with other features. By developing a method to identify such related fingerprint features, we studied their effects on analysis of compound similarity. Analyzing fingerprint feature matrices in the datasets of human metabolites and drug compounds, we found that commonly used predfined 2D structure fingerprint schemes had many related fingerprints and these fingerprints affected the scoring of structural similarity differently depending on compound pairs, which could bias the outcome of similar compound rankings and qualitatively change the list of potential hits. Interestingly, our analysis showed that the presence of related fingerprints had a general trend to mildly yet consistently lower the Tanimoto coefficient and these negative effects were seen to be substantial for a subset of compound pairs.

Previously, we developed a method to predict thermodynamic parameters of biochemical reactions by using predefined 2D chemical fingerprints and chemical descriptors as features [19]. In this regression problem, we dealt with high degrees of multicollinearity in the fingerprint-based features and sought to enhance the generalization ability using a LASSO-based feature selection and a regularized linear regression model. Here, to analyze the effects of features with a high degree of multicollinearity on search for similar compounds, we considered a different approach that computes eigenvalue-based entropy to identify related 2D fingerprint features regardless of their ability to classify similar compounds. Because this eigenvalue-based entropy approach is an unsupervised method, it is expected to be integrated seamlessly to exiting similarity-based VS pipelines that use 2D fingerprints as feature vectors.

Our results indicate that the presence of related fingerprints in predefined fingerprint dictionaries can pose challenges in objectively ranking the degree of similarity among compounds from different datasets and for different query compounds, making the evaluation of structural similarity for not only multiple-molecule queries but also single-molecule queries difficult. As each compound dataset can have different sets of related fingerprints, our results demonstrate the importance of knowing which fingerprints have high degrees of relatedness and how such related features affect the Tanimoto similarity scores. This study also emphasizes that an increase in the number of structural fingerprints may not always enhance the search performance for similar compounds and that feature selection is a valuable preprocessing step for the task of SAR analysis.

## Data Availability

The code and data used in this study have been uploaded to GitHub and are available at https://github.com/hkuwahara/chem-fp-lambda-entropy.

## References

[1] Smith A (2002). Screening for drug discovery: the leading question. Nature.

[2] Lyne PD (2002). Structure-based virtual screening: an overview. Drug Discovery Today.

[3] Willett P (2006). Similarity-based virtual screening using 2D fingerprints. Drug Discovery Today.

[4] Scior T, Bender A, Tresadern G, Medina-Franco JL, Martínez-Mayorga K (2012). Recognizing pitfalls in virtual screening: a critical review. J Chemical Information Modeling.

[5] Cereto-Massagué A, Ojeda MJ, Valls C, Mulero M, Garcia-Vallvé S (2015). Molecular fingerprint similarity search in virtual screening. Methods.

[6] Durant JL, Leland BA, Henry DR, Nourse JG (2002). Reoptimization of MDL keys for use in drug discovery. J Chemical Information Computer Sci.

[7] Mellor CL, Marchese Robinson RL, Benigni R, Ebbrell D, Enoch SJ (2019). Molecular fingerprint-derived similarity measures for toxicological read-across: Recommendations for optimal use. Regulatory Toxicol Pharmacol.

[8] Bender A, Mussa HY, Glen RC, Reiling S (2004). Molecular similarity searching using atom environments, information-based feature selection, and a naïve bayesian classifier. J Chemical Information Computer Sci.

[9] Geppert H, Vogt M, Bajorath J (2010). Current trends in ligand-based virtual screening: molecular representations, data mining methods, new application areas, and performance evaluation. J Chemical Information Modeling.

[10] Heikamp K, Bajorath J (2011). How do 2D fingerprints detect structurally diverse active compounds? Revealing compound subset-specific fingerprint features through systematic selection. J Chemical Information Modeling.

[11] Nisius B, Vogt M, Bajorath J (2009). Development of a fingerprint reduction approach for Bayesian similarity searching based on Kullback-Leibler divergence analysis. J Chemical Information Modeling.

[12] Alter O, Brown PO, Botstein D (2000). Singular value decomposition for genome-wide expression data processing and modeling. Proceedings of the National Academy of Sciences of the United States of America.

[13] Varshavsky R, Gottlieb A, Linial M, Horn D (2006). Novel unsupervised feature filtering of biological data. Bioinformatics (Oxford, England).

[14] Wishart DS, Feunang YD, Marcu A, Guo AC, Liang K (2018). HMDB 4.0: the human metabolome database for 2018. Nucleic Acids Res.

[15] Wishart DS, Feunang YD, Guo AC, Lo EJ, Marcu A (2018). Drugbank 5.0: a major update to the drugbank database for 2018. Nucleic Acids Res.

[16] Willighagen EL, Mayfield JW, Alvarsson J, Berg A, Carlsson L (2017). The chemistry development kit (cdk) v2.0: atom typing, depiction, molecular formulas, and substructure searching. J Cheminformatics.

[17] Knox C, Law V, Jewison T, Liu P, Ly S (2011). DrugBank 3.0: a comprehensive resource for ‘omics’ research on drugs. Nucleic Acids Res.

[18] Franco P, Porta N, Holliday JD, Willett P (2014). The use of 2d fingerprint methods to support the assessment of structural similarity in orphan drug legislation. J Cheminformatics.

[19] Alazmi M, Kuwahara H, Soufan O, Ding L, Gao X (2019). Systematic selection of chemical fingerprint features improves the Gibbs energy prediction of biochemical reactions. Bioinformatics.

